# Supplementary material for: Repeated Stereotactic Radiotherapy for Local Brain Metastases Failure or Distant Brain Recurrent: A Retrospective Study of 184 Patients
Source: Cancers (Basel). 2023 Oct 11;15(20):4948. doi: 10.3390/cancers15204948 (PMC10605441; doi:10.3390/cancers15204948)
Supplement: Supplementary file 1 [file cancers-15-04948-s001.zip › cancers-2550295-supplementary.pdf]

Supplementary files

Figure S1. Overall survival (a) Function of primary tumor ; (b) Function of synchronous extracerebral metastases ; (c) Function of presence of systemic treatment at SRT1 ; (d) Function of WBRT

(a) Function of primary tumor

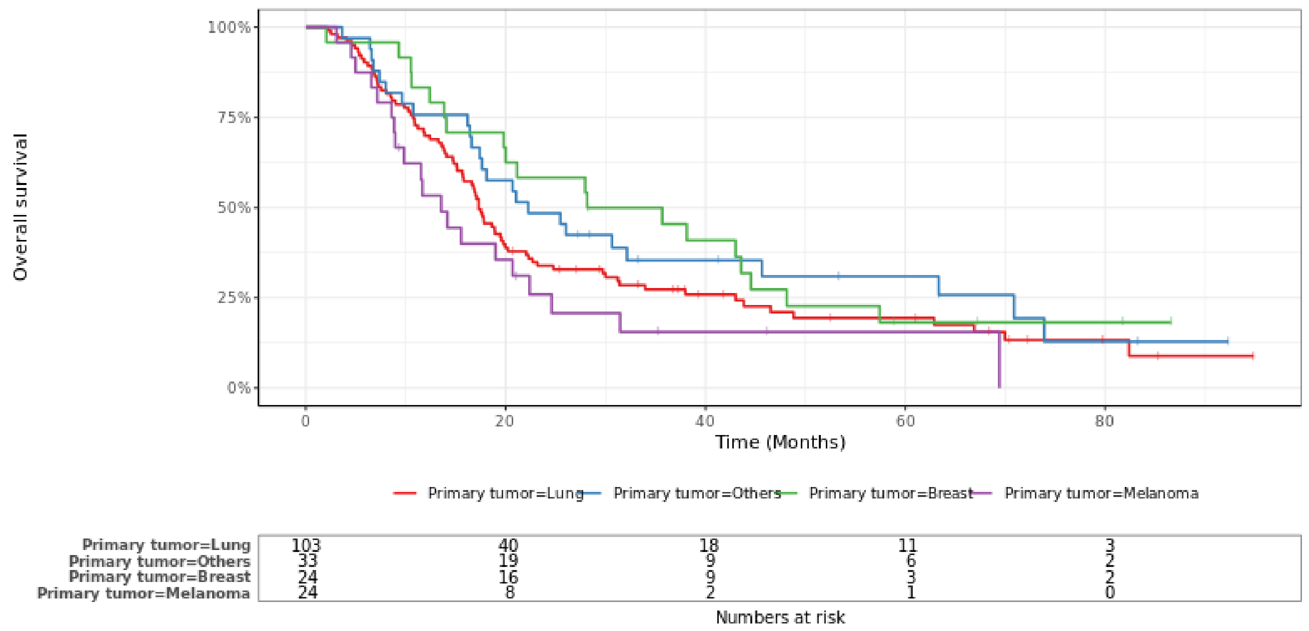

(b) Function of synchronous extracerebral metastases

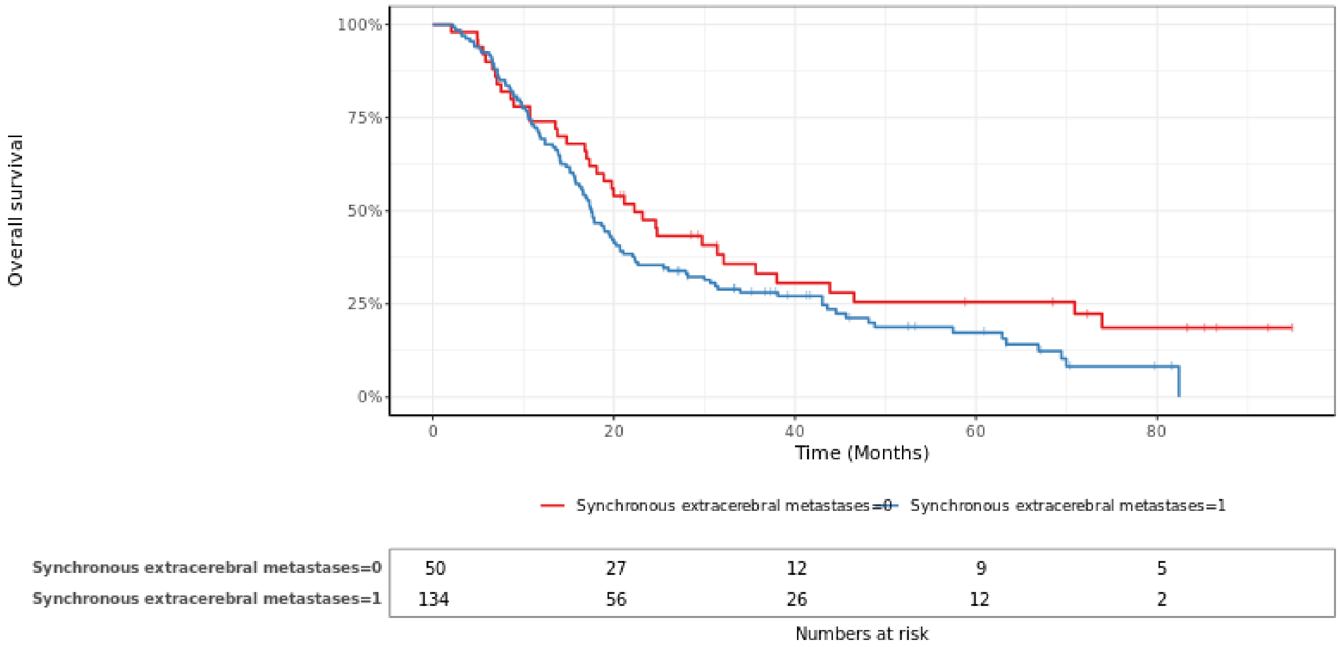

(c) Function of presence of systemic treatment at SRT1

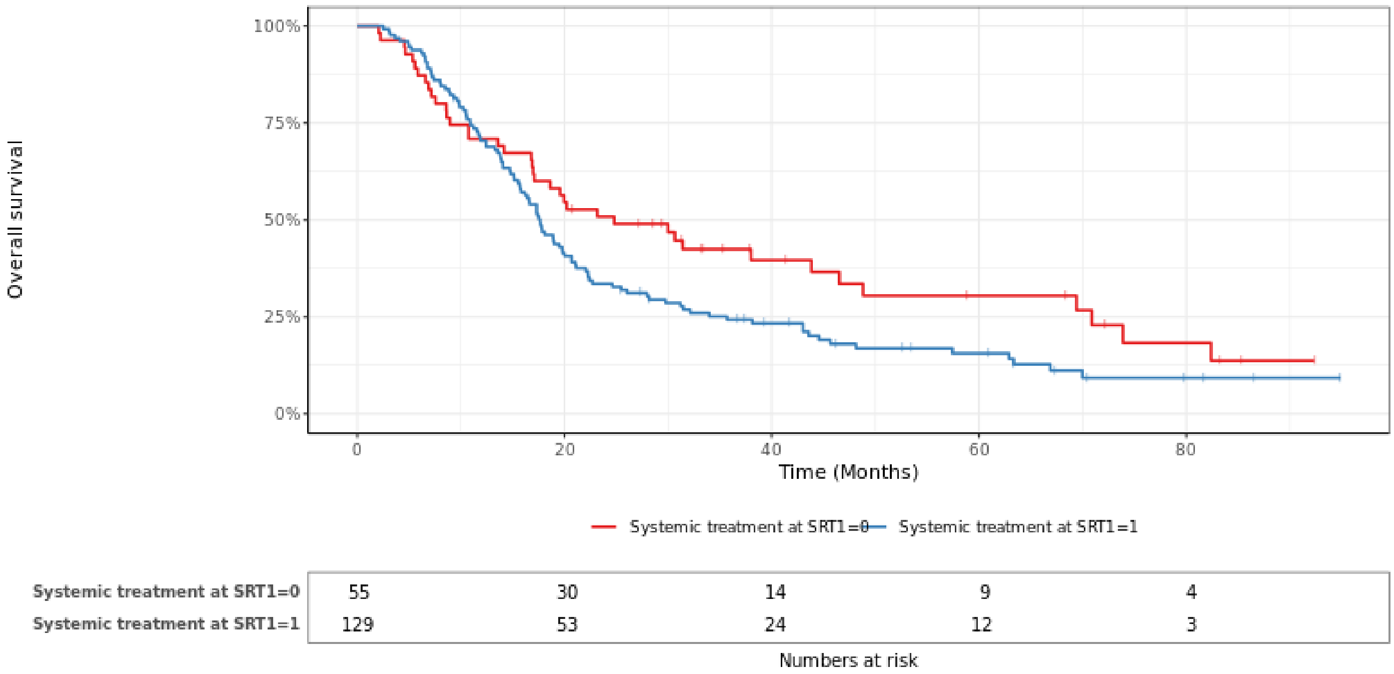

(d) Function of WBRT

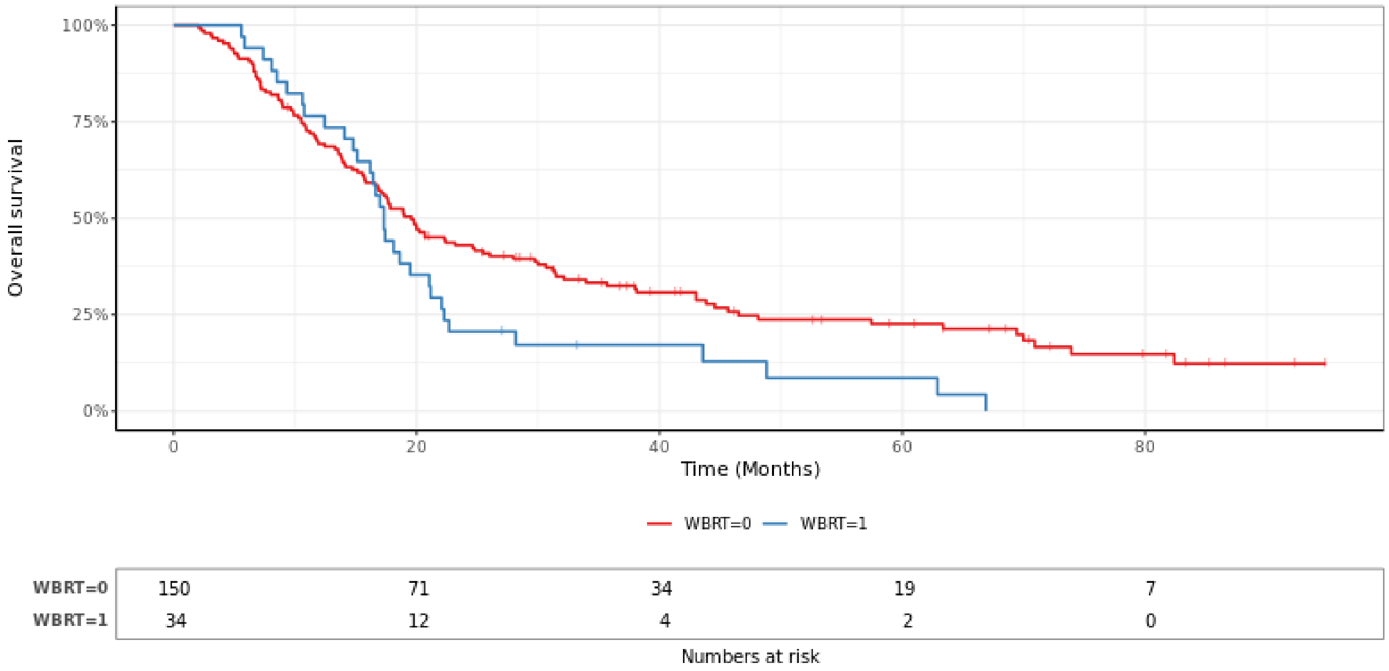

Figure S2. Neurologic-Death Free Survival (a) Function of number of SRT session; (b) Function of BMV grade; (c) Function of salvage treatment

(a) Function of number of SRT session

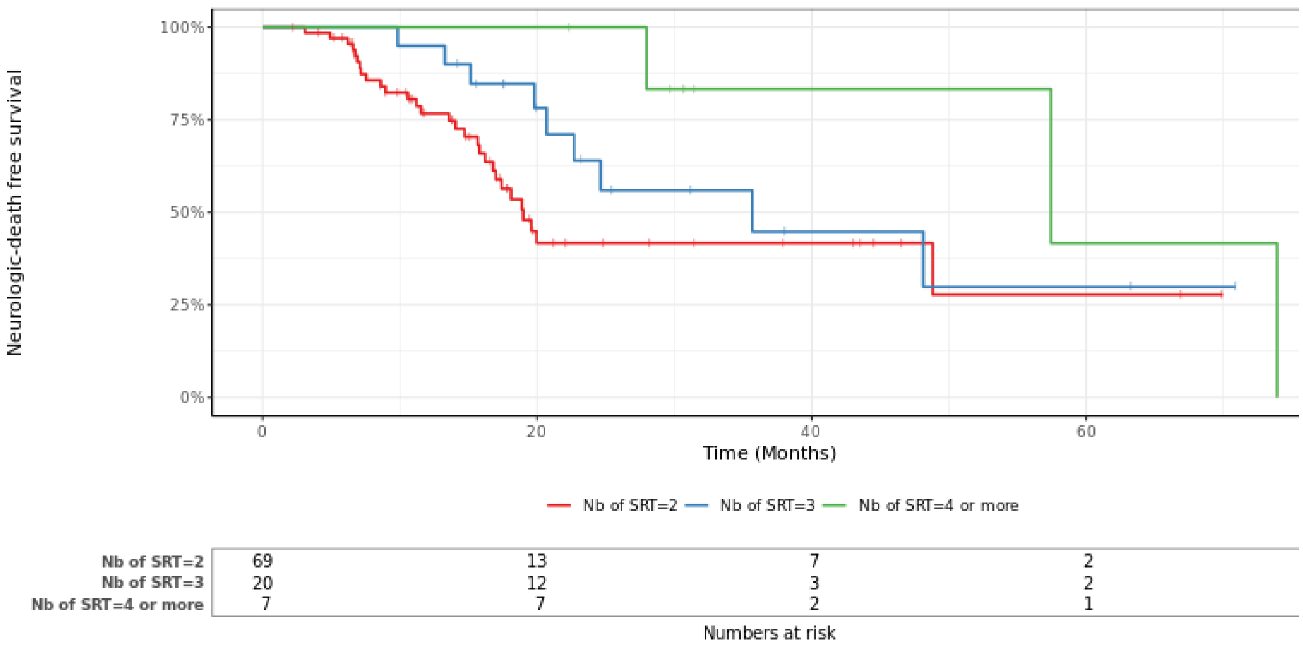

(b)

(b) Function of BMV grade

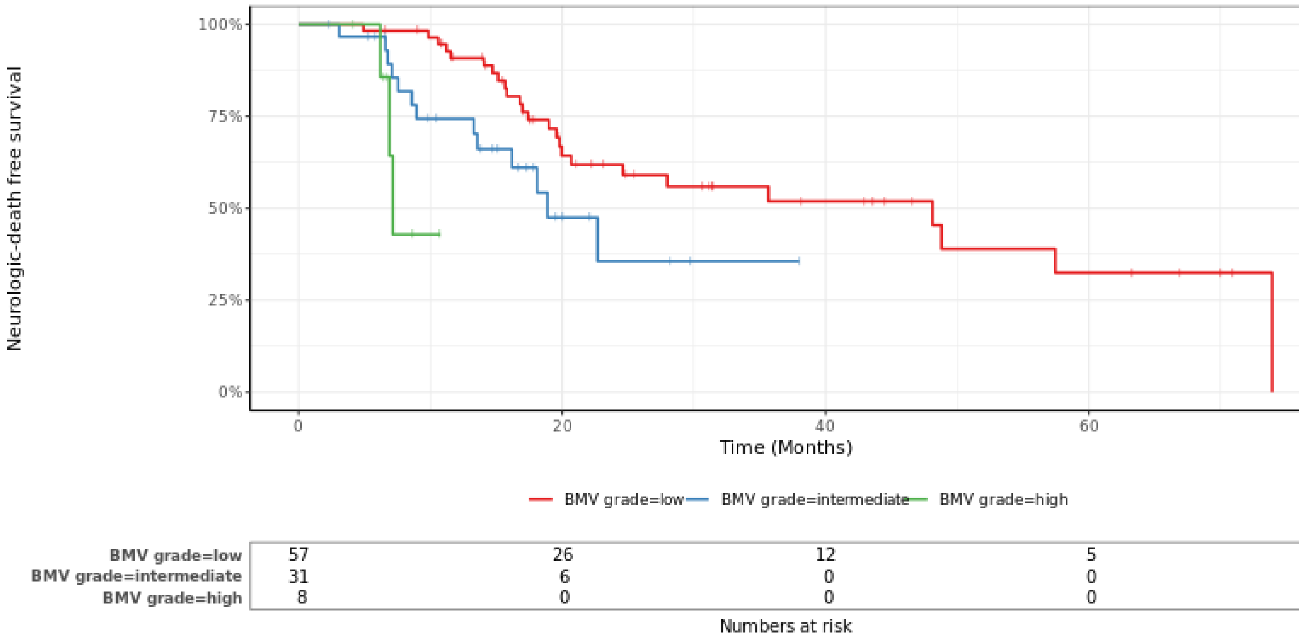

(c)

(c) Function of salvage treatment

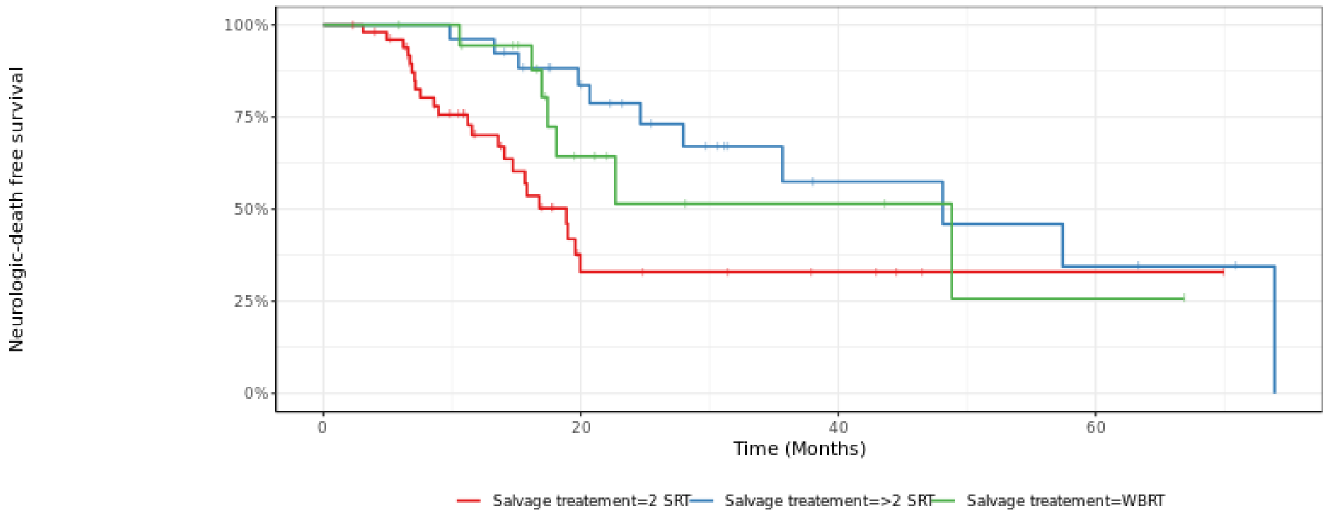

**Salvage treatement=2 SRT**	51	7	4	1
**Salvage treatement=>2 SRT**	26	18	5	3
**Salvage treatement=WBRT**	19	7	3	1

Numbers at risk
